# Ecological aspects and molecular detection of *Leishmania* DNA Ross (Kinetoplastida: Trypanosomatidae) in phlebotomine sandflies (Diptera: Psychodidae) in terra firme and várzea environments in the Middle Solimões Region, Amazonas State, Brazil

**DOI:** 10.1186/s13071-015-0789-2

**Published:** 2015-03-25

**Authors:** Antonio Marques Pereira Júnior, Carolina Bioni Garcia Teles, Ana Paula de Azevedo dos Santos, Moreno de Souza Rodrigues, Eric Fabrício Marialva, Felipe Arley Costa Pessoa, Jansen Fernandes Medeiros

**Affiliations:** Instituto Nacional de Pesquisas da Amazônia/Fiocruz Rondônia, Porto Velho, Rondônia Brazil; Laboratório de Ecologia de Doenças Transmissíveis da Amazônia (EDTA) – Centro de Pesquisa Leônidas e Maria Deane – Fiocruz Amazônia, Manaus, Amazonas Brazil; Centro de Pesquisa Gonçalo Moniz - Fiocruz, Salvador, Bahia Brazil; Laboratório de Entomologia - Fiocruz Rondônia, Porto Velho, Rondônia, Porto Velho, Rondônia Brazil

**Keywords:** Diversity, Amazon environments, Cutaneous Leishmaniasis, Richness, Diversity, Vectors

## Abstract

**Background:**

Phlebotomine sand flies (Diptera: Psychodidae) are insects of medical importance due to the role that some species play in the transmission of leishmaniasis. This work aimed to study some ecological aspects among sand flies fauna inhabiting two different environments: the várzea (lowland Amazonian forest) and terra firme (upland Amazonian forest), both located in Tefé Municipality, Amazonas State, Braziland to detect *Leishmania* infection in those phlebotomine populations.

**Methods:**

Sand flies were collected using HP light traps. Collection took place over the course of six months: January, February, April, August, September, and October of 2013. To detect natural infection by *Leishmania*, DNA samples were extracted from female sand flies and submitted to Polymerase Chain Reaction (PCR) targeting the kDNA gene; *Leishmania* species were identified by PCR-RFLP targeting the hsp70 gene and genetic sequencing.

**Results:**

In all, 5,716 individuals were collected, and 46 species were identified. *Trichophoromyia ubiquitalis* (3,330 – 58.26%) and *Nyssomyia antunesi* (661 – 11.26%) were the most abundant species. Species richness was greater in terra firme environments (42 species) than in the várzea environments (22 species), and forests ecotopes (43 species) were richer than peridomiciles (28 species). DNA of *Leishmania* was found in *Th. ubiquitalis* and *Psychodopygus davisi*, both of which inhabit the terra firme environment and sequencing analysis confirmed the presence of *Leishmania (Viannia) lainsoni* DNA in *Th. ubiquitalis* in Tefé Municipality*.*

**Conclusions:**

The high abundance of *Th. ubiquitalis* and *Ps. davisi* and detection of DNA of *Leishmania* sp. may indicate that both species could be putative vectors for American Cutaneous Leishmaniasis (ACL) in the terra firme environment of Tefé. The sand fly fauna found in várzea is rich and diverse, exhibiting several species, nevertheless the seasonal hydric stress during part of the year that could influence the local diversity, if compared with other studies. This is the first report in Amazonas State of *Th. ubiquitalis* with presence of *L. (V.) lainsoni* DNA.

## Background

American Cutaneous Leishmaniasis (ACL) is an illness characterized by single or multiple skin lesions. In Brazil, there are seven species of *Leishmania* that cause the disease [1]. Approximately 148.315 cases were reported between 2007 and 2013 [2]. In Amazonas, the largest state in the North Region of Brazil, approximately 12.727 cases were reported during the same period; these were cases related to: *Leishmania (Leishmania) amazonensis* Vianna*, L. (Viannia) braziliensis* Vianna*, L. (V.) guyanensis* Floch, and *L. (V.) naiffi* Lainson and Shaw [3].

Human cases of ACL in the region result from direct contact between humans and female phlebotomine sand flies that are hematophagous, and transmit *Leishmania* by bite. These insects are mainly found in primary forests [4,5]. However, recent studies indicate that sand flies have adapted to disturbed environments, such as forest fragments, and peridomicile areas where domestic animals are present (dogs, chickens, pigs) [6-8]. Such close proximity to human habitation increases the risk of ACL infection. Approximately 260 species of phlebotomine sand flies are found in Brazil [9]; of these, 135 species are found in Amazonas [7,10-12]. Amazonas State has the highest concentration ACL vectors these include: *Bichomomyia flaviscutellata* (Mangabeira)*, Bi. olmeca nociva* (Young and Arias), *Ny. anduzei* (Rozeboom), *Ny. antunesi* (Coutinho), *Ny. umbratilis* (Ward e Fraiha), and *Trichophoromyia ubiquitalis* (Mangabeira) [6,8,10].

In the Amazon basin, several human activities involve direct contact between humans and ACL vectors; these include: agriculture, timber harvest, suburb development, and, recently, the clearing of routes for oil pipelines [13-15]. In Amazonas, these activities mostly take place in two environments: the várzea and terra firme forests. Terra firme forest is characterized by permanently non-flooded areas, with 30 meter high trees and a high diversity of flora and fauna. Várzea forest is also rich in flora and fauna, however, unlike terra firme forest, it is flooded annually by white water rivers [16,17].

Phlebotomine sand flies have been well documented in Amazonas, particularly in terra firme environments. However, past research has been concerned primarily with Manaus and its neighboring localities. Information from other regions of Amazonas is scarce. In particular, phlebotomine sand flies that inhabit várzea environments have not been well documented, since few studies have been undertaken.

The Middle Solimões river basin contains both terra firme and várzea forests. Phlebotomine sand fly studies conducted in this region indicate that the most abundant species are of the genera *Psychodopygus, Nyssomyia* and *Trichophoromyia*; these genera include species considered to be ACL vectors [18,19]. In addition, cases of ACL in this region have increased considerably in recent years due to the presence of oil and gas extraction companies [20]. In Tefé Municipality, 328 cases of ACL were reported between 2007 and 2013, which is equivalent to 10.93 cases per 100,000 habitants, however, information about the species of *Leishmania* circulating in humans is unavailable [2]. In spite of the increased incidence of ACL in this region, few entomological studies have been undertaken to identify possible leishmaniasis vectors.

In addition, information about the natural infection of sand flies indicates that unconfirmed vectors may be present in Amazonas. Most natural infection studies in the region have been performed by dissection and visualization of trypanosomatid forms, but this method complicates the identification of *Leishmania* species [5,21,22], and is considered a relatively laborious technical procedure. Molecular methods like Polymerase Chain Reaction (PCR) are able to detect minimal amounts of *Leishmania* DNA, and allow a larger number of sand flies to be analyzed [23]; however, in Amazonas, PCR method to detect *Leishmania* infection has been used in a single article [24]. Our study aimed to study and compare the abundance and diversity of sand fly fauna in várzea and terra firme environments, and to detect *Leishmania* DNA in sand flies in an area of endemic ACL.

## Methods

**Figure 1:**
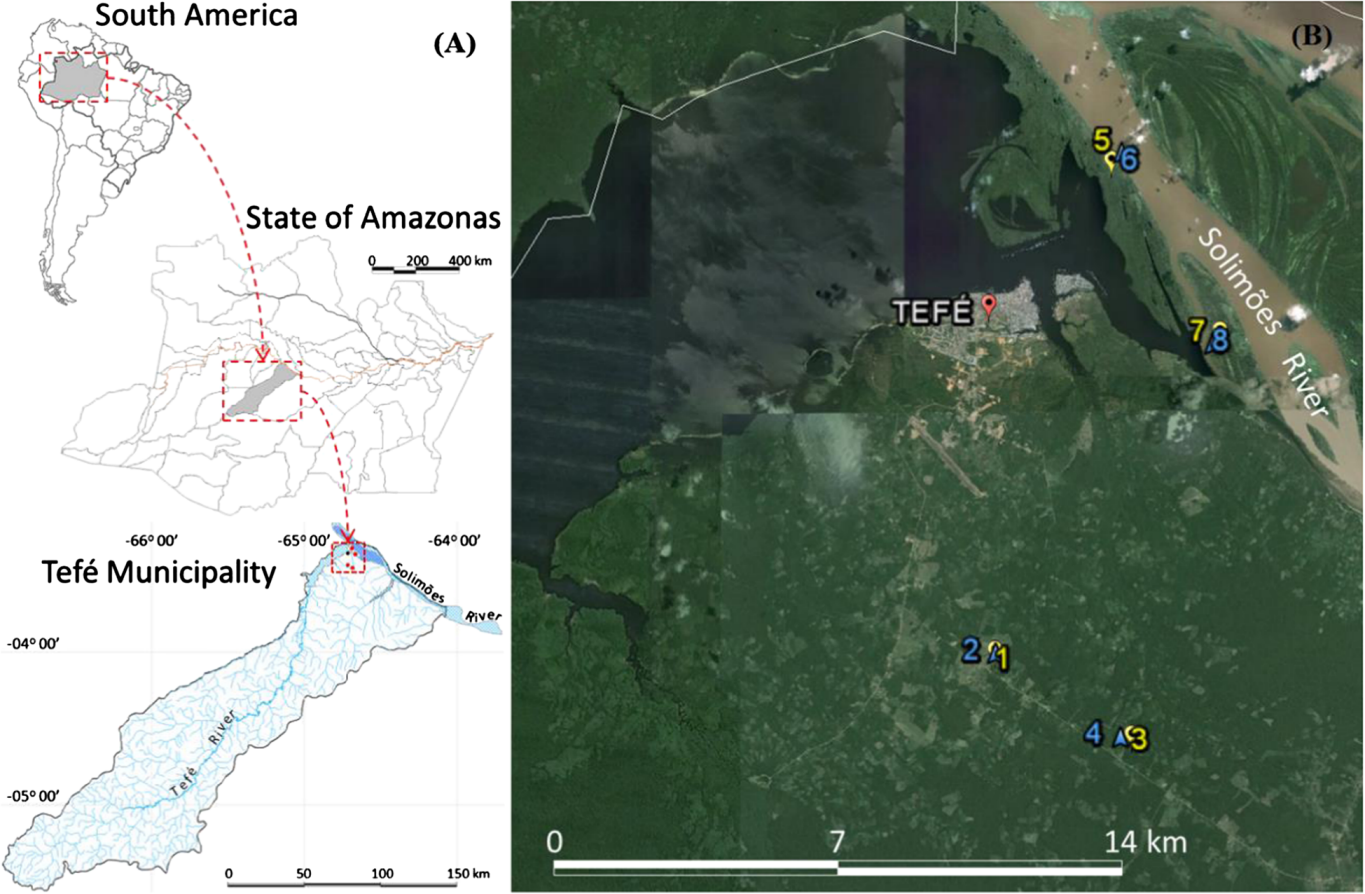
**Map of study area. (A)** Location of collection area in Tefé Municipality, Brazil. **(B)** Sand fly sampling areas in terra firme environments (1 and 2: Km 03; 3 and 4: Km 08) and várzea environments (5 and 6: Nossa Senhora do Perpétuo Socorro community; 7 and 8: Porto Vale community), Tefé Municipality, Amazonas State, Brazil. Numbers in yellow indicate forest ecotopes, and numbers in blue indicate peridomicile ecotopes. Source: Google Earth.

The Tefé Municipality (03°21’05”S, 64°42’53”W) is located in the middle of Amazonas State (AM), Brazil. It is one of ten municipalities that comprise the Middle Solimões region (Figure 1A). It has an area 23,704.488 km^2^, and a population of 61,453 habitants [25]. The climate is classified as Afi in the Köppen classification scheme [26]. The main vegetation consists of dense ombrophylous forest lowlands and alluvial ombrophylous forests [27].

Phlebotomine sand flies were collected in forest and peridomicile areas, near small farms, in both terra firme and várzea environments. Collection took place in the following localities: EMADE road (Km 03 and Km 08), the community of Nossa Senhora do Perpétuo Socorro community, and the community of Porto Vale (Figure 1B). Forest and peridomicile areas were selected and analyzed in each locality. Collections were made with HP light traps over eight consecutive nights (from 6:00 pm to 7:00 am). Collections were conducted in January, February, April, August, September, and October of 2013. The collection of sand flies was authorized by Insttuto Nacional do Meio Ambiente (IBAMA) register: 5864300, protocol number: 41382.

### Sand fly processing

After each capture event, sand flies were conserved in 90% alcohol and taken to a laboratory. Collected males, and the head and genitalia of females were clarified in Potassium Hydroxide solution and mounted on slides with Berlese fluid. All specimens were identified by taxonomic keys [28,29]. The taxonomical nomenclature of Galati [28] was used with the generic abbreviations proposed by Marcondes [30]. The rest of the body of females were conserved in tubes containing 90% alcohol in order to assess the posteriors for natural infection by *Leishmania* sp.

#### DNA Extraction of female sand flies and parasites

The females were grouped in pools of 10–20 specimens. Pools were divided according to month, environment, and species. The pools were processed for DNA extraction using the protocols for Blood and Tissue Qiagen DNeasy®. Samples were stored at -20° C for use in Polymerized Chain Reaction (PCR) testing.

#### PCR by kDNA

PCR targeting the kinetoplast region of *Leishmania* (PCR mkDNA) was performed using primers: Forward 5’-GGG (GT) AGGGGCGTTCT (G/C) CGAA-3’and Reverse 5-‘(G/C) (G/C) (G/C) (A/T) CTAT (A/T) TTACACCAACCCC-3’ to amplify the 120 bp fragment. The reaction mixture contained 18.7 μL of ultrapure water, 2.5 μL Buffer, 0.75 MgCl_2_ (2 mM), 0.38 μL prime rs (1μmol), 0.50 dNTPs (0.2 mM), 0.25 Taq Polymerase Invitrogen (1.25 U), and 2 μL of extracted DNA, for a total volume of 25 μL. Thermal cycling conditions followed those of Oliveira et al. [31].

#### PCR RFLP hsp70 and DNA sequencing

PCR targeting the hsp70 region of *Leishmania* species was performed with primers: Forward 5’-GGACGAGATCGAGCGATGGT-3’ and Reverse 5’- TCCTTCGACGCCTCCTGGTTG-3’ to amplify the 240 bp fragment. The reaction mixture contained 36.25 μL of ultrapure water, 5.0 μL Buffer, 1.5 MgCl_2_ (2 mM), 2 μL primers (1 μmol), 2 μL dNTPs (0.2 mM), 0.5 Taq Polymerase Invitrogen (1.25 U), and 5 μL of extracted DNA, for a total volume of 52.25 μL. The samples were placed in a thermocycler (Veriti–Applied Biossystems®) with an initial denaturation of 94°C for 4 minutes, followed by 33 cycles, of 94°C for 15 seconds (denaturation), 58°C for 30 seconds (annealing), and 72°C for 30 seconds (extension), with a final extension of 72°C for 10 minutes [32]. DNA from male sand flies was used as a negative control for each PCR. The final products amplified to 240 bp were submitted to restriction fragment length polymorphism (RFLP) by enzyme *HaeIII* (Invitrogen®, USA), according to manufacturer instructions.

The amplified products were visualized on a 100 bp ladder after electrophoresis using a 12% polyacrylamide gel colored with silver nitrate. The following DNA reference strains were used as positive controls: *L. (L.) amazonensis* (IOCL 575), *L. (V.) braziliensis* (IOCL 566), *L. (V.) guyanensis* (IOCL 565), *L. (V.) lainsoni* (IOCL 1045), *L. (V.) naiffi* (IOCL 1365), and *L. (V.) shawi* (IOCL 1545), obtained from Coleção de *Leishmania* do Instituto Oswaldo Cruz – Fiocruz/Rio de Janeiro, Brazil.

*Leishmania* species were identified by comparing the sequences obtained from analysis with reference sequences deposited in GenBank. Comparisons were made using BLAST program searches (Basic Local Alignment Search Tool, NCBI. Available online from: http://blast.ncbi.nlm.nih.gov.

#### Statistical analysis

The ranked abundance distribution (RAD) was calculated. The RAD produced a “hollow curve,” indicating that communities contain a few abundant species and many rare species [33]. The function radfit fit all models to a data frame, and the Akaike Information Criterion (AIC) was used to select the best one for the community. An analysis of variance (ANOVA) was performed to compare the number of species collected from each environment (i.e. Terra Firme and Várzea). The specificity and fidelity of sand fly species to environments was verified using the indicator value (IndVal) of species [34]; the morphotypes were excluded from this test. All analysis was done in R program, with a 5% significance level [35]. Natural infection was assessed by estimating the prevalence of infection in positive pools using Pool Screening Program (http://www.soph.uab.edu/bst/poolscreen) Version 2.0 [36]. Analysis of prevalence has been used previously to assess natural infection in Simuliidae [36], and the use of Pool Screening Program to assess sand flies has been suggested by Mártin-Sánchez et al. [37].

## Results

### Fauna composition of sand flies

Of the 5,716 individuals collected, 46 species were identified, including 6 morphotypes belonging to the following 11 genera: *Evandromyia* Mangabeira*, Lutzomyia* França, *Micropygomyia* Barretto*, Nyssomyia* Barretto*, Pintomyia* Costa Lima*, Psathyromyia* Barretto*, Psychodopygus* Mangabeira*, Sciopemyia* Barretto*, Trichophoromyia* Barretto*, Trichopygomyia* Barretto, and *Viannamyia* Mangabeira. The most abundant genera were *Psathyromyia* (8), *Psychodopygus* (7), *Evandromyia* (5), and *Trichophoromyia* (5). The most abundant species were *Trichophoromyia ubiquitalis, Nyssomyia antunesi*, and *Nyssomyia yuilli yuilli* (Table 1).

**Table 1:** **Phlebotomine sand flies collected in terra firme and várzea environments, Tefé Municipality, Amazonas State, Brazil**

**Species**	**Várzea**				**Terra firme**				**Várzea total**	**Terra firme total**	**Total**	**%**
	**Forest**		**Peridomicile**		**Forest**		**Peridomicile**					
	♂	♀	♂	♀	♂	♀	**♂**	♀				
*Thichophoromyia ubiquitalis* ^*(a)*^	9	6	3	0	1827	1157	257	71	18	3312	3330	58.26
*Nyssomyia antunesi* ^*(a)*^	43	79	3	10	62	186	46	232	135	526	661	11.56
*Nyssomyia yuilli yuilli*	0	2	0	0	0	249	0	10	2	259	261	4.57
*Psychodopygus davisi* ^*(a)*^	1	0	0	0	127	76	2	2	1	207	208	3.64
*Trichophoromyia* sp.	0	4	0	0	0	183	0	15	4	198	202	3.53
*Evandromyia walkeri*	11	67	2	7	9	27	27	11	87	74	161	2.82
*Trichophoromyia melloi*	0	0	0	0	123	0	23	0	0	146	146	2.55
*Psychodopygus amazonensis*	2	0	0	0	45	20	1	0	2	66	68	1.19
*Sciopemyia sordellii*	0	0	0	0	5	48	1	9	0	63	63	1.10
*Psychodopygus h. hirsutus* ^*(a)*^	0	0	0	0	0	55	0	1	0	56	56	0.98
*Psychodopygus ayrozai* ^*(a)*^	0	0	0	0	14	33	0	3	0	50	50	0.87
*Thrichophoromyia flochi*	0	0	0	0	44	0	1	0	0	45	45	0.79
*Psathyromyia dendrophyla*	0	1	0	0	36	4	1	0	1	41	42	0.73
*Psychodopygus claustrei*	0	0	0	0	10	25	1	0	0	36	36	0.63
*Viannamyia tuberculata*	0	2	0	0	0	34	0	0	2	34	36	0.63
*Lutzomyia marinkellei*	0	0	0	0	0	33	0	2	0	35	35	0.61
*Psathyromyia scaffi*	5	1	0	0	9	16	2	2	6	29	35	0.61
*Lutzomyia falcata*	0	0	0	0	0	31	0	1	0	32	32	0.56
*Sciopemyia preclara*	0	1	0	0	14	12	0	0	1	26	27	0.47
*Evandromyia begonae*	0	0	0	0	0	24	0	2	0	26	26	0.45
*Psathyromyia aragaoi*	0	1	0	0	12	7	0	0	1	19	20	0.35
*Trichopygomyia rondoniensis*	0	0	0	0	18	0	0	0	0	18	18	0.31
*Psychodopygus* sp.	0	2	0	0	0	16	0	0	2	16	18	0.31
*Evandromyia tarapacaensis*	0	0	0	0	2	4	1	6	0	13	13	0.23
*Psathyromyia souzacastroi*	5	0	0	0	6	0	1	0	5	7	12	0.21
*Nyssomyia anduzei* ^*(a)*^	1	1	0	0	7	1	0	1	2	9	11	0.19
*Nyssomyia umbratilis* ^*(a)*^	0	0	0	0	6	5	0	0	0	11	11	0.19
*Trichopygomyia* sp.	0	1	0	0	0	9	0	1	1	10	11	0.19
*Psathyromyia runoides*	0	0	0	0	0	7	1	2	0	10	10	0.17
*Psathyromyia shannoni*	2	0	0	0	7	1	0	0	2	8	10	0.17
*Pintomyia serrana*	4	2	0	0	1	0	0	1	6	2	8	0.14
*Viannamyia furcata*	0	0	0	0	7	0	1	0	0	8	8	0.14
*Lutzomyia sherlocki*	0	0	0	0	0	5	0	0	0	5	5	0.09
*Viannamyia caprina*	0	1	0	0	1	3	0	0	1	4	5	0.07
*Micropygomyia chassignetti*	0	0	0	0	4	0	0	0	0	4	4	0.07
*Trichopygomyia wagleyi*	0	0	0	0	4	0	0	0	0	4	4	0.07
*Micropygomyia micropyga*	1	2	0	0	0	0	0	0	3	0	3	0.05
*Psathyromyia campbelli*	0	2	0	0	1	0	0	0	2	1	3	0,05
*Psychopdopygus llanosmartinsi*	0	0	0	0	3	0	0	0	0	3	3	0,05
*Psathyromyia lutziana*	0	0	0	0	0	2	0	1	0	3	3	0,05
*Psathyromyia* sp.	0	0	0	0	0	3	0	0	0	3	3	0,05
*Evandromyia saulensis*	0	1	0	0	0	1	0	0	1	1	2	0,03
*Psychodopygus paraensis*	0	0	0	0	0	2	0	0	0	2	2	0,03
*Trichopygomyia dunhami*	1	0	0	0	1	0	0	0	1	1	2	0,03
*Evandromyia bourrouli*	1	0	0	0	0	0	0	0	1	0	1	0,02
*Evandromyia* sp.	0	0	0	0	0	1	0	0	0	1	1	0,02
*Lutzomyia* sp.	0	0	0	0	0	1	0	0	0	1	1	0,02
*Micropygomyia pilosa*	0	0	0	0	0	1	0	0	0	1	1	0,02
*Micropygomyia rorotaensis*	0	0	0	0	1	0	0	0	0	1	1	0,02
*Trichophoromyia auraensis* ^*(a)*^	1	0	0	0	0	0	0	0	1	0	1	0,02
*Trichopygomyia longispina*	0	0	0	0	1	0	0	0	0	1	1	0,02
**Total**	**87**	**176**	**8**	**17**	**2407**	**2282**	**366**	**373**	**288**	**5428**	**5716**	**100**

^(a)^Species considered vectors or naturally infected by flagellates in Brazil [69].

**Figure 2:**
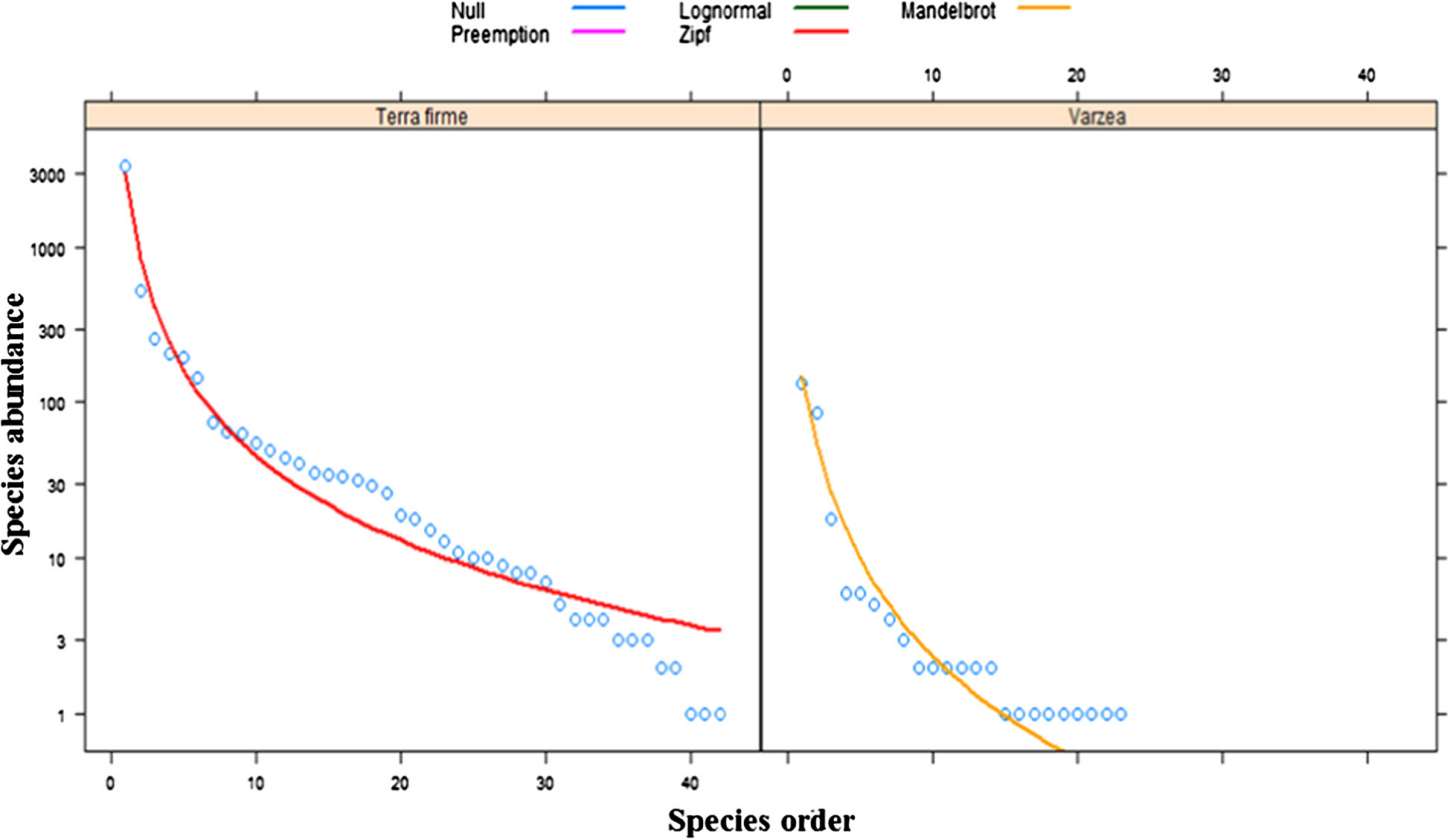
**Abundance curves.** Sand flies species in terra firme and várzea environments, captured in the months of January, February, April, August, September and October of 2013, in Tefé municipality, Amazonas State, Brazil.

In all, 5428 specimens were collected from terra firme environments, comprising 42 species and 6 morphotypes. The most abundant species were *Thichophoromyia ubiquitalis* (3312 individuals – 57.94%), *Nyssomyia antunesi* (526 – 9.20%), *Nyssomyia yuilli yuilli* (259 – 4.53%), *Trichophoromyia* sp*.* (207 – 3.62%), *Psychodopygus davisi* (198 – 3.46%), and *Trichophoromyia melloi* (146 - 2.55%). In the várzea environment, 288 specimens were collected, comprising 22 species and 3 morphotypes. Of these, the most abundant species were *Ny. antunesi* (135 - 2.36%) and *Evandromyia walkeri* (87 – 1.52%) (Table 1). Both environments had high species richness, but equitability was low (Figure 2). Analysis by IndVal indicated that only four species were specific to the terra firme environment: *Th. ubiquitalis*, *Ps. davisi*, *Ny. yuilli yuilli*, and *Th. melloi* (Table 2). No species were specific to the várzea environment, but *Evandromyia walkeri* was found there in higher frequency.

**Table 2:** **Individual Value of species from várzea and terra firme environments, Tefé municipality, Amazonas state, Brazil**

**Species**	**Environment**	**IndVal (%)**	**P**	**Frequency**
*Trichophoromyia ubiquitalis*	Terra Firme	90.33	0.001	82
*Psychodopygus davisi*	Terra Firme	48.97	0.001	40
*Nyssomyia yuilli yuilli*	Terra Firme	48.74	0.001	40
*Trichophoromyia melloi*	Terra Firme	44.30	0.001	35
*Evandromyia walkeri*	Várzea	35.71	0.01	53

**Figure 3:**
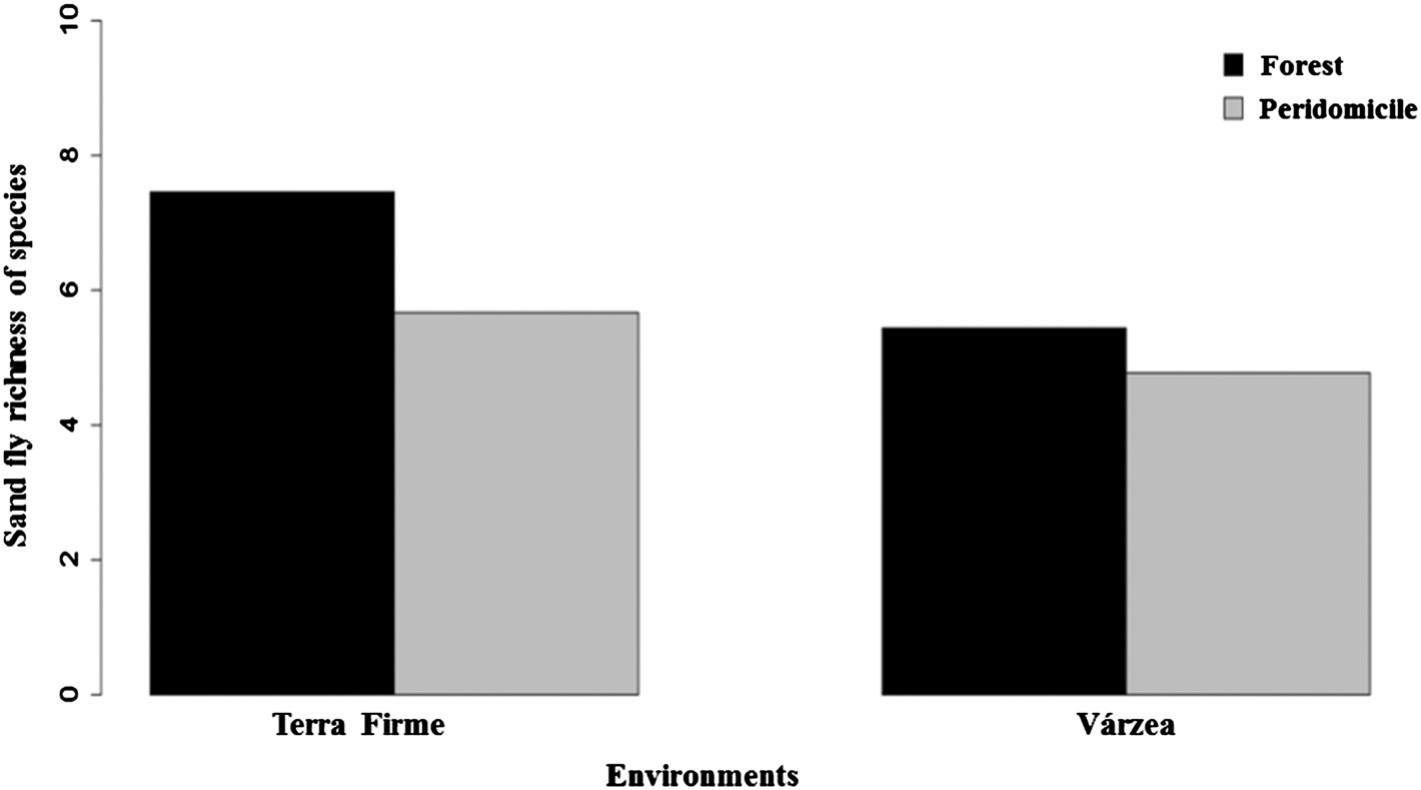
**Richness of sand flies.** Richness level in peridomicile (grey) and forest (black) ecotopes of the terra firme and várzea environments, Tefé municipality, Amazonas State, Brazil.

Sand fly richness was highest in the forest ecotopes of both environments as compared with peridomiciles (ANOVA *χ*^2^ = 3.95, p < 0.04) (Figure 3). In the terra firme environment, forest ecotopes yielded 4689 specimens and 39 species, while peridomiciles ecotopes yielded 739 individuals and 27 species. In the várzea environment, forest ecotopes yielded 263 individuals and 21 species, while peridomicile ecotopes yielded 25 individuals and 4 species (Table 1).

#### Detection of *Leishmania* DNA in phlebotomine sand flies

**Figure 4:**
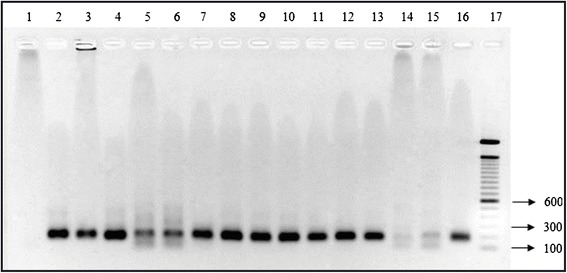
**PCR mKDNA.** 2% agarosis gel colored with GelRed showingproducts amplified by kDNA PCR with DNA extracted from female sand flies collected in Tefé Municipality, Amazonas State, Brazil. Lines: 1 – negative control (Ultrapure water - H2O Milli-Q); 2 to 4 - *Thichophoromyia ubiquitalis*; 5 to 6 - *Psychodopygus davisi*; 7 to 13: *Trichophoromyia ubiquitalis*; 14 to 15: *Psychodopygus davisi*; 16: positive control *Leishmania (Leishmania) amazonensis,* 17: molecular marker 100pb (Invitrogen).

In total, 1,679 females (58.9% of all females collected) were grouped in 95 pools (*Th. ubiquitalis* – 60 pools, *Ny. antunesi* – 18, *Ny. yuilli yuilli* – 8, *Ps. davisi* – 4, *Ps. ayrozai* – 4, *Ny. umbratilis* - 1). Of these, 14 pools were amplified in the mkDNA region and assessed as DNA *Leishmania* presence confirmed: *Th. ubiquitalis* - 10 pools, and *Ps. davisi –* 4 pools. Both of these species were specific to the terra firme environment (Figure 4). The minimal infection prevalence for *Th. ubiquitalis* was 0. 96% (95% CI = 0.51–**0.96–**0.17) and all samples of *Ps. davisi* tested positive for infection.

**Figure 5:**
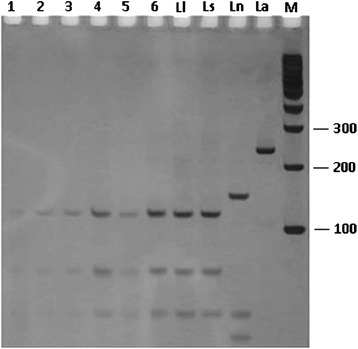
**PCR-RFLP (234 bp) profiles for samples of** ***Trichophoromyia ubiquitalis*** **after disgestion of products with** ***Hae*** **III.** Silver-stained 12.5% polyacrylamide gel. Lines: 1 - pool 65; 2 - pool 4; 3 - pool 24; 4 - pool 70; 5 - pool 72; 6 - pool 77; 7 – Ll = *Leishmania (Viannia) lainsoni*; 8 – Ls = *Leishmania (Viannia) shawi;* 9 – Ln = *Leishmania (Viannia) naiffi*;10 – La = *Leishmania (Leishmania) amazonensis*; M = 100 bp molecular weight marker 100 bp (Invitrogen).

Seven samples of *Th. ubiquitalis* were amplified by PCR targeted to the hsp70 region. Restriction enzyme *Hae*III was applied to these samples yielding the patterns of *L. (V.) lainsoni* and *L. (V.) shawi* (Figure 5). Sequencing confirmed the presence of *L. (V.) lainsoni* in all samples*.* The sequences for pool 70_hsp70 and pool 72_hsp70 were similar to the sequence of *L. (V.) lainsoni* (99%) deposited in GenBank under code number GU071176.1.

## Discussion

The species found in Tefé Municipality correspond to 34.5% of the species recorded in Amazonas [7,10-12]. Phlebotomine fauna in the Middle Solimões region is rich and diverse, being comprised of 40–50 species; of which, *Th. ubiquitalis*, *Ny. yuilli yuilli*, *Ps. chagasi*, and *Ps. davisi* are the most abundant [18,19]. Also Silva et al. [24] found *Th. ubiquitalis* and *Ps. davisi* as a dominant species in Lábrea Municipality, nearby Purus River, a Solimões River tributary. Those species composition is different from the species composition found in Manaus and its neighboring municipalities, where there are 35–50 species, the most abundant being *Ny. umbratilis*, *Ny. anduzei*, and *Ny. antunesi*. This shows that sand fly fauna differs throughout Amazonas State [5-8,38,39].

A high diversity of genera was found in Tefé, the highest were respectively *Psathyromyia*, *Psyhodopygus*, and *Trichophoromyia*, which are widely distributed throughout the Amazon region, and are also found in others states of Brazil [10,40-44]. These genera are of epidemiological importance because they are present in several regions of Brazil, and they contain some species involved or suspected in the transmission of ACL and other etiological agents [1].

There is a scarcity of data that compares sand fly fauna in terra firme and várzea environments simultaneously. However, phlebotomine fauna in Tefé is considered diverse in both environments, (31.1% in Terra firme and 16.3% in Várzea if compared with a total of species recorded to Amazonas). Some species incriminated or suspected as vectors have been recorded in the terra firme environment: *Ny. anduzei, Ny. antunesi, Ny. umbratilis, Ps. davisi*, *Ps. h. hirsutus*, and *Th. ubiquitalis*. This highlights the possibility that these species may participate in the transmission of ACL in both of the environments studied in Tefé. Some studies of fauna in the várzea environment have identified insects of medical importance, such as mosquitoes and biting midges [45-47]; however, there is little information about sand flies [48]. This is because the várzea environment is difficult to access during rainy seasons, and because flooding makes field work more arduous; yet flooding may have a direct impact on the habitats of insect fauna. Our results show that fauna in the várzea environment is rich, exhibiting several species such as *Ny. antunesi, Th. auraensis*, and *Th. ubiquitalis*, that are of medical importance and could pose epidemiological concerns [1,49].

In Tefé, Forest ecotopes proved to be richer and more abundant than peridomicile ecotopes. Sand flies are found mainly in forest environments probably because the resources there are more plentiful. The abundance observed in this study has been observed in other studies in the Amazon region, and these studies have found that most species reside in forest environments [8,50]. However, abundance has been observed in some species residing in localities with anthropic effects. These species include *Th. ubiquitalis, Ev. walkeri,* and *Ny. antunesi*, and this shows some adaptation to modified environments [7,8,51]*.* These species may have been attracted by blood meal sources such as humans, or pigs and chickens present in peridomicile areas [52-54].

*Trichophoromyia ubiquitalis* was the most abundant species in this study. This species is abundant in other municipalities and is widely distributed throughout the Amazon region, including the Middle Solimões region and tributaries [18,19,24], and the municipalities of Borba and Maués, near to Manaus [10]. This species also occurs in high abundance in the rest of the North Region, being found in the states of Acre, Pará, and Rondônia [44,55-57]. Recently, Silva et al. [24] found the presence of *L. (L.) amazonensis* DNA in *Th. ubiquitalis* in Lábrea Municipality, Amazonas. The *Nyssomyia antunesi* was the second most abundant species in this study. This species has adapted to anthropogenic environments and has been recorded as abundant in the municipalities of Lábrea, and Presidente Figueiredo, in Amazonas [7,8]. This species has been found naturally infected by *L. (V.) lindenbergi* in Pará State [58], and has been linked to the transmission of ACL in that region.

The detection of *Th. ubiquitalis* and *Ps. davisi* with *Leishmania* DNA could be an indicative of those species are putative vectors in Tefé. Previously, the presence of DNA *L.(L.) amazonensis* and *L. (V.) braziliensis* were detected respectively in the species *Th. ubiquitalis* and *Ps. davisi* [24]. Although *Th. ubiquitalis* exhibits low anthropophilic behavior, it is a proven vector of *L. (V.) lainsoni* in Pará State [57]. *Psychodopygus davisi* has been found infected by *Leishmania* spp. in Rondônia State [55,59], and naturally infected by *L. (V.) braziliensis* in Pará State, in Serra do Carajás, and in Peru [49,60] and with *Leishmania braziliensis* DNA presence in Lábrea [24]. The minimal infection prevalence of 0.96% observed in *Th. ubiquitalis* does not differ from that of other studies, where a 0.3–3% incidence of natural infection has been considered high [61-66].

This is the first record in Amazonas State of sand flies with presence of *L. (V.) lainsoni* DNA. The only previous record of this *Leishmania* species in the Amazonas State was a human case involving the infection of a single child in Manaus [67]. The distribution of *L. (V.) lainsoni* has probably been underestimated, as its presence in Brazil has only been attributed to the states of Acre, Pará [57,68], and Amazonas. The detection of DNA clew of *L. (V.) lainsoni* in *Th. ubiquitalis*, and the high abundance of this fly may indicate that this species could be a putative vector in In Tefé Municipality.

## Conclusion

In the Middle Solimões region, the sand fly fauna in terra firme and várzea environments is composed of a few dominant species, and several species with few individuals. The fauna varies between ecotopes, being more abundant in forest ecotopes than in peridomicile ecotopes. The abundance of *Th. ubiquitalis* and its record of presence of *L. (V.) lainsoni* DNA may indicate that this species is a vector for ACL in Tefé Municipality, Amazonas, Brazil.

## Abbreviations

ACL: American cutaneous leishmaniasis
PCR: Polymerase chain reaction
DNA: Deoxyribonucleic acid
HP: Hoover pugedo
kDNA: Kinetoplatisc DNA
hsp70: Heat shock protein 70
bp: Base pairs
RAD: Ranked abundance distribution
AIC: Akaike information criterion
ANOVA: Analyzes of variance
INDVAL: Indicator value of species
Ll: *Leishmania lainsoni*
Ls: *Leishmania shawi*
Ln: *Leishmania naiffi*
La: *Leishmania amazonensis*
